# Transcriptomic profiling of human hippocampal progenitor cells treated with antidepressants and its application in drug repositioning

**DOI:** 10.1177/0269881117691467

**Published:** 2017-02-16

**Authors:** Timothy R Powell, Tytus Murphy, Sang H Lee, Jack Price, Sandrine Thuret, Gerome Breen

**Affiliations:** 1 Social, Genetic and Developmental Psychiatry, Institute of Psychiatry, Psychology and Neuroscience, King’s College London, London, UK; 2 National Institute for Health Research Biomedical Research Centre for Mental Health, Institute of Psychiatry, Psychology and Neuroscience, Maudsley Hospital and King’s College London, London, UK; 3 Department of Basic and Clinical Neuroscience, Institute of Psychiatry, Psychology and Neuroscience, King’s College London, London, UK

**Keywords:** Antidepressants, connectivity mapping, neural stem cells, major depression, drug repositioning

## Abstract

Current pharmacological treatments for major depressive disorder (MDD) are ineffective in a significant proportion of patients, and the identification of new antidepressant compounds has been difficult. ‘Connectivity mapping’ is a method that can be used to identify drugs that elicit similar downstream effects on mRNA levels when compared to current treatments, and thus may point towards possible repositioning opportunities. We investigated genome-wide transcriptomic changes to human hippocampal progenitor cells treated with therapeutically relevant concentrations of a tricyclic antidepressant (nortriptyline) and a selective serotonin reuptake inhibitor (escitalopram). We identified mRNA changes common to both drugs to create an ‘antidepressant mRNA signature’. We used this signature to probe the Library of Integrated Network-based Cellular Signatures (LINCS) and to identify other compounds that elicit similar changes to mRNA in neural progenitor cells. Results from LINCS revealed that the tricyclic antidepressant clomipramine elicited mRNA changes most similar to our mRNA signature, and we identified W-7 and vorinostat as functionally relevant drug candidates, which may have repositioning potential. Our results are encouraging and represent the first attempt to use connectivity mapping for drug repositioning in MDD.

## Introduction

Major depressive disorder (MDD) is a complex disorder characterised by a pathological distortion of affect (Jones et al., 2002). Antidepressants are the first line treatment for clinical MDD, but they are ineffective in a significant proportion of patients (Gibson et al., 2010). Evidence suggests that antidepressant drugs of different classes may evoke some convergent downstream effects at the messenger RNA (mRNA) level (Malki et al., 2012). Genetic factors can further moderate how initial upstream targets within each drug class relay their effects on to downstream transcriptional pathways (Malki et al., 2011; Powell et al., 2013). Consequently, increasing the number of available compounds with differing upstream mechanisms, but convergent downstream effects, will increase our chances of effectively treating the disorder in patients from different genetic backgrounds.

Identifying new types of antidepressant compounds, however, has been slow, with most being discovered through serendipity (Ban, 2006). Drawing from other areas of medicine, one way to identify new therapeutic compounds is through drug repositioning; the application of known drugs and compounds to new indications (Ashburn et al., 2004). There has been some promising evidence of drug repositioning in other fields of medicine using ‘connectivity mapping’, facilitated by the use of the Library of Integrated Network-based Cellular Signatures (LINCS; Lamb et al., 2006; Vempati et al., 2014). LINCS is essentially a mRNA library characterising the effects of over 20,000 small-molecule compounds, including over 1300 FDA approved drugs, in cell lines (Qu and Rajpal, 2012). LINCS allows users to identify compounds that elicit similar or opposite mRNA profiles to an experimentally-derived query mRNA signature (Qu and Rajpal, 2012).

Promising lines of research indicate that the hippocampus, and more specifically, hippocampal progenitor cells, are a key target of antidepressant medications (Boldrini et al., 2009; Malberg et al., 2000). Indeed, recent research suggests that antidepressant treatment in proliferating human hippocampal progenitor cells primes them for a route of advanced differentiation into neurons (antidepressant-induced hippocampal neurogenesis), which may be important in mediating their therapeutic effects (Anacker et al., 2011). Furthermore, in the rare cases where potentially new antidepressant compounds have been identified (e.g. the melatonergic analogue, agomelatine), they too seem to enhance neuroplasticity and neurogenesis in the hippocampus (Pompili et al., 2013).

This study attempts to tackle three main aims. First, we aim to investigate the effects of two antidepressants from different classes, a selective serotonin reuptake inhibitor (SSRI; escitalopram) and a tricyclic antidepressant (nortriptyline), on mRNA levels in human hippocampal progenitor cells. Second, we aim to isolate the overlapping effects of both drugs on gene expression, to create an ‘antidepressant mRNA signature’. Third we aim to use this antidepressant mRNA signature to probe the LINCS database and search for other compounds that elicit similar (and opposite) effects on mRNA to our signature, specifically in neural progenitor cells. The identification of known antidepressant compounds amongst those showing similar mRNA signatures to our query signature would confirm we have identified functionally meaningful mRNA targets. Furthermore, the identification of other functionally relevant compounds may point towards novel repositioning opportunities for MDD.

## Methods

### The hippocampal progenitor cell line

The multipotent, human hippocampal progenitor cell line HPC0A07/03C (provided by ReNeuron, Surrey, UK) was used for all experiments, as described previously (Anacker et al., 2011). ReNeuron’s HPC0A07/03C cells were obtained from a 12-week old foetus and immortalised with c-mycER technology. In the presence of growth factors (FGF2 and EGF) and 4-OHT, progenitors cells proliferate indefinitely. Cells were grown in Dulbecco’s Modified Eagle’s Media/F12 (DMEM:F12, Invitrogen, Paisley, UK) supplemented with 0.03% human albumin (Baxter Healthcare, Compton, UK), 100 µg/mL human apo-transferrin (Sigma, St Louis, MO, USA), 16.2 µg/mL human putrescine DiHCl (Sigma), 5 µg/mL human insulin (Sigma), 60 ng/mL progesterone (Sigma), 2 mM l-glutamine (Sigma) and 40 ng/mL sodium selenite (Sigma), 10 ng/mL human bFGF (Pepro Tech EC Ltd, London, UK), 20 ng/mL human EGF (Pepro Tech EC Ltd) and 100 nM 4-OHT (Sigma). The cell line underwent routine checking for mycoplasma contamination every 6 weeks. All cells were grown at 37°C, 5% CO_2_, and in a humidified atmosphere. Within these sets of experiments each ‘biological replicate’ represents a subculture of cells obtained from a different passage.

### Drug doses

The active metabolite of the SSRI escitalopram, (*S*)-citalopram, has a therapeutic window of between 50–130 ng/mL in serum, which corresponds to doses of between 120–313 nM *in vitro*. The active metabolite of the tricyclic antidepressant nortriptyline, 10-hydroxynortriptyline, has a therapeutic window of between 70–170 ng/mL, which corresponds to 233–567 nM *in vitro*. Subsequently, cells were treated with a range of doses, incorporating two therapeutically-relevant doses of each drug and a high drug dose group comparable to those used previously in this cell line (Anacker et al., 2011).

Escitalopram drug doses were achieved by dissolving escitalopram oxalate (Sigma) in molecular grade ethanol (Sigma) to form a 10 mM stock solution. Escitalopram drug doses (0 nM, 145 nM, 290 nM and 1160 nM) were then formed by dilution of the stock with media, with the relative proportion of ethanol kept constant across all dose groups. Nortriptyline drug doses were achieved by dissolving nortriptyline hydrochloride (Sigma) in RNase free water to form a 10 mM stock solution. Nortriptyline drug doses (0 nM, 267 nM, 534 nM, 1068 nM) were then formed by dilution of the stock in media. To confirm that the drug doses were not toxic, we checked for any effects on cell death using immunohistochemistry. There was no evidence of increased cell death related to any dose in either drug condition (see Supplementary Information).

### Culture protocol

Cells were seeded for 24 h on laminin-coated 6 well plates (Nunclon, Roskilde, Denmark). After seeding, cells were treated with media containing varying doses of escitalopram oxalate or nortriptyline hydrochloride for 48 h. At the end of the 48-h drug treatment period, proliferating media was aspirated and 1 ml of Trireagent (Sigma) was added for RNA isolation. Our 48-h drug culture duration represents a midrange between the treatment durations of neural progenitor cells in LINCS (6, 24 and 96 h).

### Transcriptomics

RNA from cell experiments was isolated using Trireagent following the standard protocol, with an additional ethanol precipitation step to increase RNA purity. For gene expression experiments we utilised six biological replicates at four doses for each drug (total *n* = 24 per drug group). RNA samples were processed on Illumina Human HT-12 v4 Expression BeadChip (Illumina Inc., San Diego, CA) according to manufacturer’s protocol, see Supplementary Information.

### Statistical analysis

Probes were normalised using a systematic approach incorporating the Lumi package (Du et al., 2008) and analysed using R (www.r-project.org). Probes were filtered to remove lowly detected probes and to investigate only the 7500 most variable probes (see Supplementary information online). We focused on the dose-dependent effects of each drug on gene expression. A linear regression was performed, where probe expression was selected as the dependent variable, dose as the independent variable, and batch and biological replicate included as factors. The false discovery rate (FDR) of multiple testing corrections was applied to our dataset with a *q*-value threshold of *q* < 0.05.

### Gene ontology analysis

To understand which biological mechanisms may be affected by drug dose in a hypothesis-free manner we input *p*-values generated from our linear regressions from each of our drug groups into the Gene Ontology enRIchment anaLysis and visuaLizAtion tool (GoriLLa; http://cbl-gorilla.cs.technion.ac.il). All probes nominally affected by drug dose (*p* ⩽ 0.05) were tested for gene ontology (GO) term enrichment with biological processes (GOTERM_BP_FAT), cellular components (GOTERM_CC_FAT) and molecular function (GOTERM_MF_FAT). We utilised all probes present on the array after background correction, as our reference list.

### Gene co-expression networks

To visualise gene co-expression networks relating to top GO terms and our antidepressant mRNA signature, we used the web-based application GeneMania (genemania.org). We input any nominally-significant transcripts identified in each condition into GeneMania, and selected the *H. sapiens* option, so that the tool would specifically draw connections between the genes in our experimentally-derived gene sets using human data only.

### Connectivity mapping

Connectivity mapping is the use of genome-wide expression data from cultured human cells treated with bioactive small molecules to discover functional associations between drugs, genes and diseases through the feature of shared gene expression changes (Lamb et al., 2006). The LINCS allows us to perform connectivity mapping and identify compounds which elicit similar downstream effects (positive connectivity scores) or opposite downstream effects (negative connectivity scores) on mRNA as our antidepressants. To implement connectivity mapping, we identified nominally-significant (*p* < 0.05) downregulated and upregulated transcripts in response to escitalopram dose or nortriptyline dose, or changes common to both drug doses, and input this information into LINCS (www.lincscloud.org). We then used the output to identify compounds (specifically) that elicit similar gene expression effects in neural progenitor cells (specifically). We considered any drugs as having a connectivity score of greater or equal to ±0.75 as potential candidates for repositioning.

## Results

### Escitalopram

**Figure 1.**
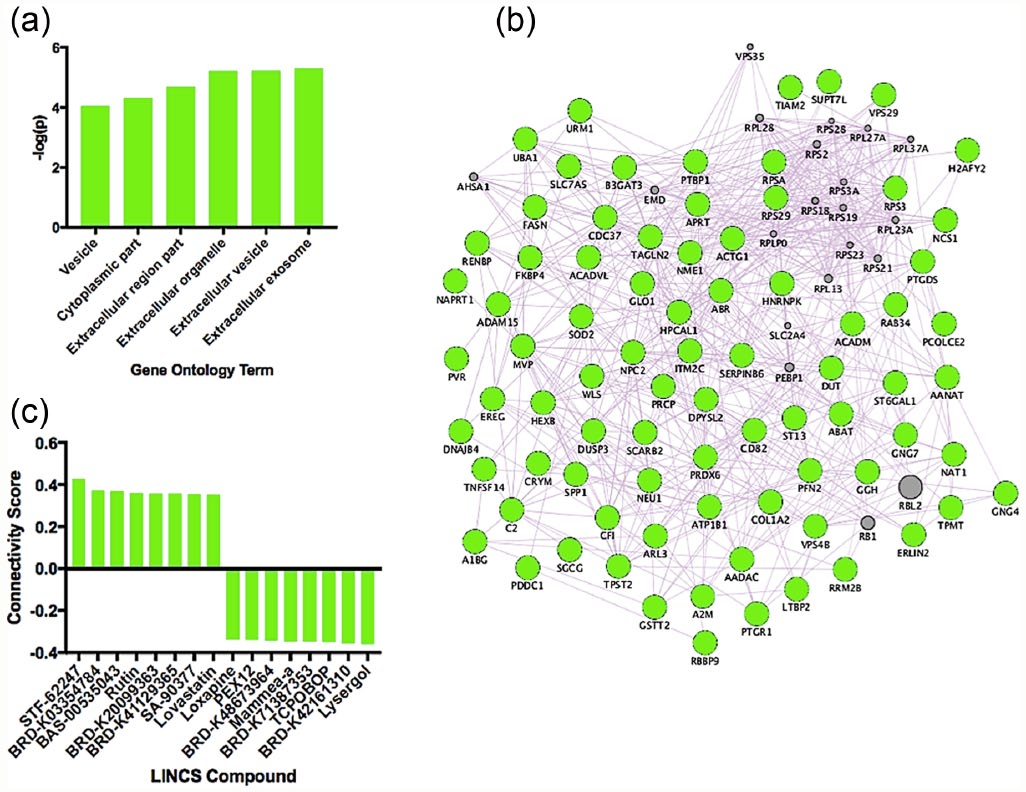
(a) Significant GO terms activated in response to escitalopram dose. (b) The top GO term identified from our data, ‘extracellular exosome’, visualised as a gene co-expression network. Genes in grey represent hypothetical transcripts connecting other genes within the network, whereas genes in green represent those transcripts identified from our dataset. (c) Eight compounds with the strongest positive connectivity scores (similar effects on mRNA) and eight compounds with the strongest negative connectivity scores (opposite effects on mRNA) in neural progenitor cells.

Our results revealed no FDR-significant transcripts associated with escitalopram dose, but 324 were nominally (uncorrected *p* < 0.05) affected by dose. Our results revealed six FDR-significant (*q* < 0.05) GO terms in cells treated with escitalopram (Figure 1(a)); the top GO term was ‘extracellular exosome’, visualised as a gene co-expression network in Figure 1(b). We further input upregulated and downregulated transcripts associated with escitalopram dose into the LINCS database and identified eight compounds with the strongest positive connectivity scores (similar effects on mRNA) and eight compounds with the strongest negative connectivity scores (opposite effects on mRNA) in neural progenitor cells, visualised in Figure 1(c).

### Nortriptyline

**Figure 2.**
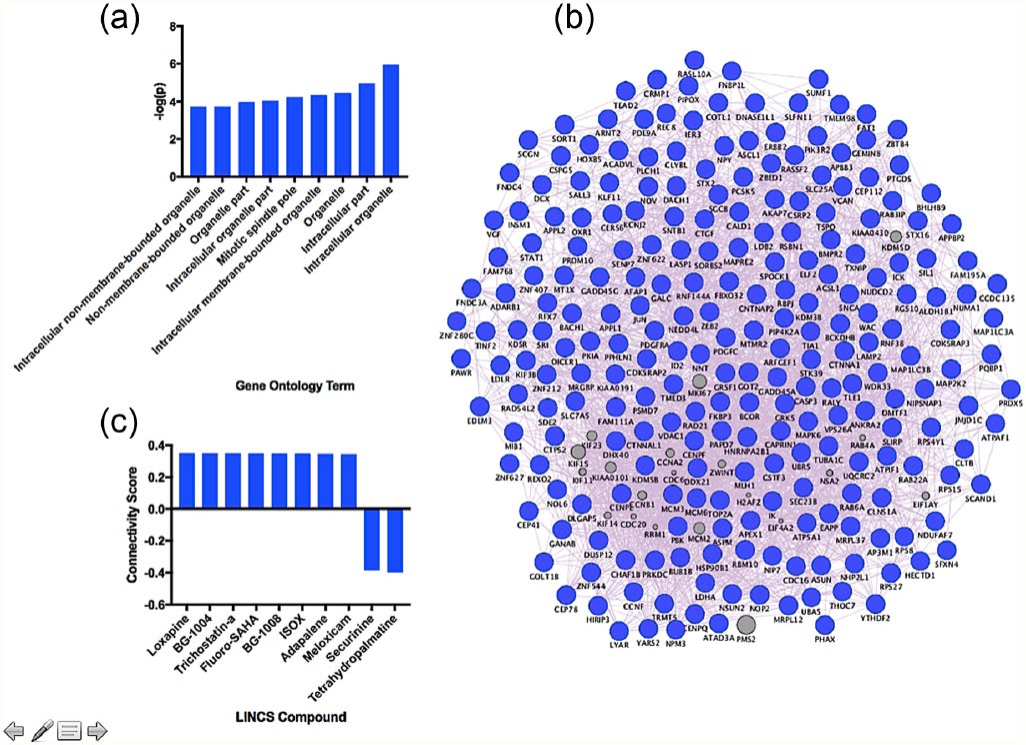
(a) Significant GO terms activated in response to nortriptyline dose. (b) The top GO term identified from our data, ‘intracellular organelle’, visualised as a gene co-expression network. Genes in grey represent hypothetical transcripts connecting other genes within the network, whereas genes in blue represent those transcripts identified from our dataset. (c) Eight compounds with the strongest positive connectivity scores (similar effects on mRNA) and two compounds with the strongest negative connectivity scores (opposite effects on mRNA; only two compounds identified) in neural progenitor cells.

Our results revealed no FDR-significant transcripts associated with nortriptyline dose, but 458 were nominally (uncorrected *p* < 0.05) affected by dose. Our results revealed nine FDR-significant (*q* < 0.05) GO terms in cells treated with nortriptyline (Figure 2(a)); the top GO term was ‘intracellular organelle’, visualised as a gene co-expression network in Figure 2(b). We further input upregulated and downregulated transcripts associated with nortriptyline dose into the LINCS database and identified eight compounds with the strongest positive connectivity scores (similar effects on mRNA) and two compounds with the strongest negative connectivity scores (opposite effects on mRNA) in neural progenitor cells, visualised in Figure 2(c).

### Overlapping effects of antidepressants

**Figure 3.**
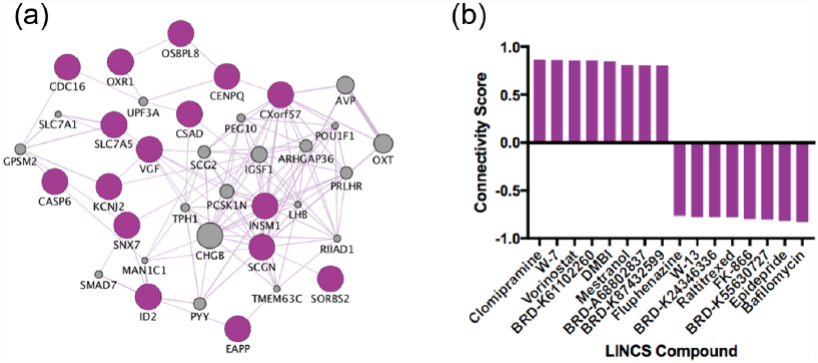
(a) Our antidepressant mRNA signature visualised as a gene co-expression network. Genes in grey represent hypothetical transcripts connecting other genes within the network, whereas genes in purple represent those transcripts identified from our dataset. (b) Eight compounds with the strongest positive connectivity scores (similar effects on mRNA) and eight compounds with the strongest negative connectivity scores (opposite effects on mRNA) in neural progenitor cells.

Thirty transcripts showed common nominally-significant changes in both drug groups (*p* < 0.05); 20 out of 30 common transcripts were in the same direction in both drug groups (11 upregulated, 9 downregulated; Table 1), which formed our ‘antidepressant mRNA signature’. The overlapping transcripts are also visualised as a gene co-expression network (Figure 3(a)), and the eight compounds with the strongest positive connectivity scores (similar effects on mRNA), and eight compounds with the strongest negative connectivity scores (opposite effects on mRNA) in neural progenitor cells, are visualised in Figure 3(b).

**Table 1.** The transcripts affected by both escitalopram and nortriptyline dose, which formed our ‘antidepressant mRNA signature’.

Direction of effect	Gene symbol	Gene name
Upregulated	CASP6	Caspase-6
	CENPQ	Centromere protein Q
	EAPP	E2F-associated phosphoprotein
	ID2	DNA-binding protein inhibitor ID-2
	INSM1	Insulinoma-associated protein 1
	MGC39900	(Hypothetical protein)
	OSBPL8	Oxysterol-binding protein-related protein 8
	OXR1	Oxidation resistance protein 1
	SCGN	Secretagogin
	SNX7	Sorting Nexin 7
	SORBS2	Sorbin and SH3 domain-containing protein 2
Downregulated	CDC16	Cell division cycle protein 16 homologue
	CSAD	Cysteine sulfinic acid decarboxylase
	CXORF57	Chromosome X open reading frame 57
	HS.25892	Small nucleolar RNA host gene 15 (non-protein coding)
	KCNJ2	Potassium voltage-gated channel subfamily J member 2
	LYAR	Cell growth-regulating nucleolar protein
	MIR1974	microRNA 1974
	SLC7A5	Solute carrier family 7 member 5
	VGF	VGF nerve growth factor inducible

## Discussion

Our results revealed that two antidepressants with differing upstream mechanisms activate a set of both distinct and common downstream molecular pathways in hippocampal progenitor cells. Our GO analysis revealed that escitalopram specifically activated gene networks relating to extracellular organelles and vesicles, suggesting that it is modulating pathways related to extracellular communication (Figure 1). However, nortriptyline primarily affected intracellular components and organelles suggesting, conversely, that it modulates components of intracellular function (Figure 2).

We used expression changes associated with antidepressant dose to probe the LINCS database and to identify compounds which could be repositioned for the treatment of MDD. Neither antidepressant alone identified compounds with very high connectivity scores; all were below ±0.75 (Figures 1 and 2), so these compounds should be considered more cautiously than those with a higher connectivity score (e.g. those identified from our antidepressant mRNA signature (Figure 3) discussed below). Identified compounds included those which were well-studied (e.g. loxapine), and relatively new compounds which may not have been tested in humans yet (e.g. BRD-K03354784), and so we will restrict our review of the suitability of compounds to those which are well-characterised.

In response to escitalopram dose, rutin was one of the top compounds with a positive connectivity score. Rutin is a citrus flavonoid found in a wide variety of plants such as citrus fruits (Davis, 1947). Numerous reports have revealed that rutin has antidepressant-like properties in mouse; for instance increasing mobility in the forced swim test (Machado et al., 2008; Nöldner and Schötz, 2002). Furthermore, it’s been identified as an essential antidepressant component to the medicinal herb, St John’s wort (*Hypericum perforatum*; Nöldner and Schötz, 2002). The compound with the highest positive connectivity score in relation to nortriptyline dose was loxapine. Loxapine is a typical antipsychotic medication used in the treatment of schizophrenia (Heel et al., 1978). Similarly to nortriptyline, loxapine has a rich pharmacology, and is metabolised by *N*-demethylation to amoxapine, a tetracyclic antidepressant (Cohen et al., 1982). Consequently, loxapine represents a compound with plausible repositioning potential for MDD.

Both escitalopram and nortriptyline are effective antidepressants and they also show common effects on mRNA levels that may represent a convergent mechanism (Table 1 and Figure 3). Overlapping transcripts include the zinc-finger transcription factor, insulinoma-associated 1 (*INSM1*), a gene which has previously been found to increase the generation and expansion of neural progenitor cells in the neocortex (Farkas et al., 2008). Therefore, this gene could be important in mediating increases in proliferation previously reported in response to antidepressants (Boldrini et al., 2012). Another gene also upregulated was oxidation resistance 1, *OXR1*, which codes for a protein that specifically protects against oxidative stress (Volkert et al., 2000). This gene may be important in mediating protection against increases in reactive oxygen species hypothesised to trigger neuronal atrophy in the brains of MDD patients (Michel et al., 2012). Consequently, both antidepressants may be activating genes important in progenitor proliferation and neuroprotection, which may be important in mediating their effects.

Despite interesting compounds being identified when considering each drug separately (described above), the main purpose of our study was to identify common effects of escitalopram and nortriptyline on mRNA (an antidepressant mRNA signature) and to identify compounds that may impinge upon these convergent molecular targets (Table 1 and Figure 3). Our results revealed compounds which evoke both reverse and similar mRNA profiles to our antidepressant mRNA signature (Figure 3). The drugs with the reverse mRNA profile represent those that hypothetically induce opposite downstream effects to antidepressants. The drug displaying the most potent reverse effects to our antidepressant mRNA signature (strongest negative connectivity score) was bafilomycin. Bafilomycin is an antibiotic which has previously been shown to reverse some of the adverse cardiac effects of antidepressants (Dennis et al., 2011). Furthermore, bafilomycin has been shown to prevent the re-acidification of synaptic vesicles in hippocampal tissue, potentially slowing the rate of neurotransmission across the synapse (Zhou et al., 2000). Therefore, functionally, bafilomycin may reverse some of the effects of antidepressants by slowing the release of neurotransmitters crossing the synapse (e.g. serotonin and noradrenaline), preventing the antidepressant’s initial mechanism of action, which is primarily to increase post-synaptic binding of serotonin and noradrenaline. However, this requires confirmation in further studies as the interactions between bafilomycin and antidepressants are not well understood, nor is it clear whether the effect of bafilomycin on synaptic vesicles is specific to the hippocampus.

Our results also revealed that the drug clomipramine had the highest connectivity score, suggesting it evokes similar changes to mRNA in neural progenitor cells as we identify in our study (Figure 3). Clomipramine is a tricyclic antidepressant used in the treatment of MDD with both serotonergic and noradrenergic effects (Amsterdam et al., 1997), and thus our results suggest that we may have identified a functionally valid set of mRNA changes, which can be used to identify compounds which elicit similar functional effects.

The drug ranked with the second highest connectivity score in the LINCS output was W-7 (*N*-(6-aminohexyl)-5-chloro-1-naphthalenesulfonamide hydrochloride). W-7 is an intracellular calcium binding protein (calmodulin) antagonist (Jan et al., 2000) and a functionally plausible compound for drug repositioning in MDD. Calmodulin antagonists and inhibition of calmodulin-dependent protein kinase II (CaMKII) disinhibit excitatory neurotransmission via presynaptic metabotropic glutamate receptors, and enhance neurotransmitter release (Hinds et al., 2003, O’Connor et al., 1999). Furthermore, antidepressants and calmodulin have both been shown to reverse corticosterone-induced gene transcription (Budziszewska et al., 2000); suggesting calmodulin inhibition reverses the downstream effects of stress, a known risk factor for MDD. Moreover, other studies have concluded that any interruption of the Ca^2+^-calmodulin-NOS-guanylyl cyclase subcellular signalling pathway will produce antidepressant-like effects (Paul, 2001).

The drug ranked with the third highest connectivity score is vorinostat, a histone deacetylase inhibitor, which has a similar mechanism of action to the mood-stabiliser sodium valproate (Kilgore et al., 2010). Both these medications are epigenetic modifiers which elicit potent effects on genome-wide gene expression (Covington et al., 2009). There is a considerable body of evidence which reveals epigenetic differences between MDD cases and controls (Saavedra et al., 2016). Furthermore, recent results from the genetic pathway analysis of an MDD genome-wide association study, suggest that genes regulating epigenetic modifications of histones might be causally related to MDD (The Network and Pathway Analysis Subgroup of the Psychiatric Genomics Consortium, 2015). Mechanistically, it could be that a genetic predisposition to MDD coincides with epigenetic modifications that result in reduced ‘transcriptional plasticity’, that impacts upon the propensity of hippocampal neural stem cells to differentiate (Burney et al., 2013). Indeed, hippocampal neural stem cell differentiation is associated with widespread transcriptional reprogramming (Mody et al., 2001), so a reduced propensity for transcriptional change may result in a reduced rate of neurogenesis, and therefore may explain the smaller hippocampal volumes observed amongst MDD patients (Bremmer et al., 2000). Moreover, histone deacetylase inhibitors such as a vorinostat, that promote transcriptional activity across the genome, have previously been found to increase neurogenesis and neuroplasticity (Grayson et al., 2010). Thus, vorinostat may represent a functionally relevant compound with the potential to be repurposed in the treatment of MDD.

Despite the promising results, this study has a number of limitations. First, we investigated the acute effects of antidepressants on gene expression profiles. However, studies suggest that chronic treatment with antidepressants may be needed in order to observe therapeutic or functional effects (e.g. Malberg et al., 2000) and consequently we may be missing important gene expression changes linked to longer culture of progenitor cells with antidepressants. Thus, the short treatment duration may result in decreased ‘construct validity’ because it doesn’t reflect the same treatment duration it takes to observe clinical change, but because 48-h corresponds closely to the treatment duration in the LINCS database, the mRNA changes we identify may have good predictive validity in the context of drug screening (Powell et al., 2012).

Secondly, we cannot, using our methodology, accurately model the interactions of drugs in an *in vivo* environment where hormones and neurotransmitter release will moderate the effects of these drugs on neural stem cells. Furthermore, we can only provide indications as to what drugs might be promising repositioning opportunities for MDD; further functional work and, ultimately, clinical trials will be needed to confirm the repositioning validity of identified compounds.

In conclusion, this study is the first to report the genome-wide effects of two antidepressants on proliferating human hippocampal progenitor cells. We identify both overlapping and distinct effects of these drugs on mRNA levels. We isolated convergent effects of two antidepressants on 20 mRNA transcripts which we used to create an antidepressant mRNA signature to probe the LINCS database. Our results identified compounds which may reverse the effects of antidepressants (bafilomycin); compounds which elicit similar changes to mRNA, and are known to elicit the same functional effects (clomipramine); and compounds which show similar mRNA effects to our antidepressants with the potential to be repositioned as novel antidepressants (W-7, vorinostat). Our findings suggest that connectivity mapping and the use of the LINCS database may have relevance in the repositioning of compounds for the treatment for MDD.

## Supplementary Material

Supplementary material

## References

[bibr1-0269881117691467] Amsterdam JD García-España F Rosenzweig M (1997) Clomipramine augmentation in treatment-resistant depression. Depress Anxiety 5: 84–90. 9262938

[bibr2-0269881117691467] Anacker C Zunszain PA Cattaneo A (2011) Antidepressants increase human hippocampal neurogenesis by activating the glucocorticoid receptor. Mol Psychiatry 16: 738–750. 21483429 10.1038/mp.2011.26 PMC3121947

[bibr3-0269881117691467] Ashburn TT Thor KB (2004) Drug repositioning: identifying and developing new uses for existing drugs. Nat Rev Drug Discov 3: 673–683. 15286734 10.1038/nrd1468

[bibr4-0269881117691467] Ban TA (2006) The role of serendipity in drug discovery. Dialogues Clin Neurosci 8: 335–344. 17117615 10.31887/DCNS.2006.8.3/tban PMC3181823

[bibr5-0269881117691467] Boldrini M Hen R Underwood MD (2012) Hippocampal angiogenesis and progenitor cell proliferation are increased with antidepressant use in major depression. Biol Psychiatry 72: 562–571. 22652019 10.1016/j.biopsych.2012.04.024 PMC3438317

[bibr6-0269881117691467] Boldrini M Underwood MD Hen R (2009) Antidepressants increase neural progenitor cells in the human hippocampus. Neuropsychopharmacology 34: 2376–2389. 19606083 10.1038/npp.2009.75 PMC2743790

[bibr7-0269881117691467] Bremmer JD Narayan M Anderson ER (2000) Hippocampal volume reduction in major depression. Am J Psychiatry 157: 115–117. 10618023 10.1176/ajp.157.1.115

[bibr8-0269881117691467] Budziszewska B Jaworska-Feil L Kajta M (2009) Antidepressant drugs inhibit glucocorticoid receptor-mediated gene transcription – a possible mechanism. Br J Pharmacol 130: 1385–1393. 10.1038/sj.bjp.0703445 PMC1572203 10903980

[bibr9-0269881117691467] Burney MJ Johnston C Wong KY (2013) An epigenetic signature of developmental potential in neural stem cells and early neurons. Stem Cells 31: 1868–1880. 23712654 10.1002/stem.1431

[bibr10-0269881117691467] Cohen BM Harris PQ Altesman RI (1982) Amoxapine: neuroleptic as well as antidepressant? Am J Psychiatry 139: 1165–1167. 6126130 10.1176/ajp.139.9.1165

[bibr11-0269881117691467] Covington HE Maze I LaPlant QC (2009) Antidepressant actions of histone deacetylase inhibitors. J Neurosci 29: 11451–11460. 19759294 10.1523/JNEUROSCI.1758-09.2009 PMC2775805

[bibr12-0269881117691467] Davis WB (1947) Determination of flavanones in citrus fruits. Anal Chem 19: 476–478.

[bibr13-0269881117691467] Dennis AT Nassal D Deschenes I (2011) Antidepressant-induced ubiquitination and degradation of the cardiac potassium channel hERG. J Biol Chem 286: 34413–34425. 21832094 10.1074/jbc.M111.254367 PMC3190784

[bibr14-0269881117691467] Du P Kibbe WA Lin SM (2008) Lumi: a pipeline for processing Illumina microarray. Bioinformatics 24: 1547–1548. 18467348 10.1093/bioinformatics/btn224

[bibr15-0269881117691467] Farkas LM Haffner C Giger T (2008) Insulinoma-associated 1 has a panneurogenic role and promotes the generation and expansion of basal progenitors in the developing mouse neocortex. Neuron 60: 40–50. 18940587 10.1016/j.neuron.2008.09.020

[bibr16-0269881117691467] Gibson TB Jing Y Smith Carls G (2010) Cost burden of treatment resistance in patients with depression. Am J Manag Care 16: 370–377. 20469957

[bibr17-0269881117691467] Grayson DR Kundakovic M Sharma RP (2010) Is there a future for histone deacetylase inhibitors in the pharmacotherapy of psychiatric disorders? Mol Pharmacol 77: 126–135. 19917878 10.1124/mol.109.061333

[bibr18-0269881117691467] Heel RC Brogden RN Speight TM (1978) Loxapine: a review of its pharmacological properties and therapeutic efficacy as an antipsychotic agent. Drugs 15: 198–217. 25167 10.2165/00003495-197815030-00002

[bibr19-0269881117691467] Hinds HL Goussakov I Nakazawa K (2003) Essential function of alpha-calcium/calmodulin-dependent protein kinase II in neurotransmitter release at a glutamatergic central synapse. Proc Natl Acad Sci USA 100: 4275–4280. 12629219 10.1073/pnas.0530202100 PMC153083

[bibr20-0269881117691467] Jan CR Yu CC Huang JK (2000) *N*-(6-Aminohexyl)-5-chloro-1-naphthalenesulfonamide hydrochloride (W-7) causes increases in intracellular free Ca^2+^ levels in bladder female transitional carcinoma (BFTC) cells. Anticancer Res 20: 4355–4359. 11205271

[bibr21-0269881117691467] Jones I Kent L Craddock N (2002) Genetics of affective disorders. In: McGuffin P Owen MJ Gottesman II (eds) Psychiatric Genetics & Genomics. New York: Oxford University Press, pp.211–245.

[bibr22-0269881117691467] Kilgore M Miller CA Fass DM (2010) Inhibitors of class 1 histone deacetylases reverse contextual memory deficits in a mouse model of Alzheimer’s disease. Neuropsychopharmacology 35: 870–880. 20010553 10.1038/npp.2009.197 PMC3055373

[bibr23-0269881117691467] Lamb J Crawford ED Peck D (2006) The connectivity map: using gene-expression signatures to connect small molecules, genes, and disease. Science 313: 1929–1935. 17008526 10.1126/science.1132939

[bibr24-0269881117691467] Machado DG Bettio LEB Cunha MP (2008) Antidepressant-like effect of rutin isolated from the ethanolic extract from Schinus molle L. in mice: evidence for the involvement of the serotonergic and noradrenergic systems. Eur J Pharmacol 587: 163–168. 18457827 10.1016/j.ejphar.2008.03.021

[bibr25-0269881117691467] Malberg JE Eisch AJ Nestler EJ (2000) Chronic antidepressant treatment increases neurogenesis in adult rat hippocampus. J Neurosci 20: 9104–9110. 11124987 10.1523/JNEUROSCI.20-24-09104.2000 PMC6773038

[bibr26-0269881117691467] Malki K Lourdusamy A Binder E (2012) Antidepressant-dependent mRNA changes in mouse associated with hippocampal neurogenesis in a mouse model of depression. Pharmacogenet Genomics 22: 765–776. 23026812 10.1097/FPC.0b013e328356fa90

[bibr27-0269881117691467] Malki K Zhou R Paya-Cano J (2011) Convergent animal and human evidence suggests a role of PPM1A gene in response to antidepressants. Biol Psychiatry 69: 360–365. 20970119 10.1016/j.biopsych.2010.08.011

[bibr28-0269881117691467] Means AR Dedman JR (1980) Calmodulin: an intracellular calcium receptor. Nature 285: 73–77. 6990273 10.1038/285073a0

[bibr29-0269881117691467] Michel TM Pülschen D Thome J (2012) The role of oxidative stress in depressive disorders. Curr Pharm Des 18: 5890–5899. 22681168 10.2174/138161212803523554

[bibr30-0269881117691467] Mody M Cao Y Cui Z (2001) Genome-wide gene expression profiles of the developing mouse hippocampus. Proc Natl Acad Sci USA 98: 8862–8867. 11438693 10.1073/pnas.141244998 PMC37526

[bibr31-0269881117691467] Nöldner M Schötz K (2002) Rutin is essential for the antidepressant activity of *Hypericum perforatum* extracts in the forced swimming test. Planta Med 68: 577–580. 12142988 10.1055/s-2002-32908

[bibr32-0269881117691467] O’Connor V El Far O Bofill-Cardona E (1999) Calmodulin dependence of presynaptic metabotropic glutamate receptor signaling. Science 286: 1180–1184. 10550060 10.1126/science.286.5442.1180

[bibr33-0269881117691467] Paul IA (2001) Antidepressant activity and calcium signaling cascades. Hum Psychopharmacol 16: 71–80. 12404601 10.1002/hup.186

[bibr34-0269881117691467] Pompili M Serafini G Innamorati M (2013) Agomelatine, a novel intriguing antidepressant option enhancing neuroplasticity: a critical review. World J Biol Psychiatry 14: 412–413. 23530731 10.3109/15622975.2013.765593

[bibr35-0269881117691467] Powell TR Fernandes C Schalkwyk LC (2012) Depression-related behavioral tests. Curr Protoc Mouse Biol 2: 119–127. 26069008 10.1002/9780470942390.mo110176

[bibr36-0269881117691467] Powell TR Schalkwyk LC Heffernan AL (2013) Tumor necrosis factor and its targets in the inflammatory cytokine pathway are identified as putative transcriptomic biomarkers for escitalopram response. Eur Neuropsychopharmacol 23: 1105–1114. 23142150 10.1016/j.euroneuro.2012.09.009

[bibr37-0269881117691467] Qu XA Rajpal DK (2012) Application of connectivity map in drug discovery and development. Drug Discov Today 7: 1289–1298. 10.1016/j.drudis.2012.07.017 22889966

[bibr38-0269881117691467] Saavedra K Molina-Márquez AM Saavedra N (2016) Epigenetic modifications of major depressive disorder. Int J Mol Sci 17: 1279. 10.3390/ijms17081279 PMC5000676 27527165

[bibr39-0269881117691467] The Network and Pathway Analysis Subgroup of the Psychiatric Genomics Consortium (2015) Psychiatric genome-wide association study analyses implicate neuronal, immune and histone pathways. Nat Neurosci 18: 199–209. 25599223 10.1038/nn.3922 PMC4378867

[bibr40-0269881117691467] Vempati UD Chung C Mader C (2014) Metadata standard and data exchange specifications to describe, model, and integrate complex and diverse high-throughput screening data from the Library of Integrated Network-based Cellular Signatures (LINCS). J Biomol Screen 19: 803–816. 24518066 10.1177/1087057114522514 PMC7723305

[bibr41-0269881117691467] Volkert MR Elliot NA Housman DE (2000) Functional genomics reveals a family of eukaryotic oxidation protection genes. Proc Natl Acad Sci USA 97: 14530–14535. 11114193 10.1073/pnas.260495897 PMC18953

[bibr42-0269881117691467] Zhou Q Petersen CCH Nicoll RA (2000) Effects of reduced vesicular filling on synaptic transmission in rat hippocampal neurones. J Physiol 525: 195–206. 10811737 10.1111/j.1469-7793.2000.t01-1-00195.x PMC2269926

