# Supplementary material for: Transcriptomic profiling of human hippocampal progenitor cells treated with antidepressants and its application in drug repositioning
Source: J Psychopharmacol. 2017 Feb 16;31(3):338–45. doi: 10.1177/0269881117691467 (PMC5349314; doi:10.1177/0269881117691467)
Supplement: Supplementary material [file JOP691467_Supplementary_material.pdf]

Supplementary Information

S1: Cell culture protocol for drug toxicity check

We selected antidepressant drug doses, which corresponded to the therapeutic ranges found in patient blood and are therefore unlikely to be toxic. However, as a precaution we checked whether antidepressant doses affected rates of cell death in our human hippocampal progenitor cell line.

To achieve this we repeated the 72-hour culture protocol described in the main text, but instead we performed this in lamin-coated 96-well plates. Three biological replicates and three technical replicates were utilised for each drug dose; meaning per drug, we had a total of 12 samples. After the completion of the 48-hour drug treatment, media was aspirated and 96-well plates were fixed in 4% paraformaldehyde for 20 min at room temperature, and then stored in phosphate buffered saline at 4°C for immunohistochemistry experiments, whereby cleaved Caspase-3 (CC3) staining was used to assay cell death.

S2: Immunohistochemistry protocol for drug toxicity check

For all wells, 50 μL of blocking solution (0.3% Triton X-100, 5% normal donkey serum (Alpha Diagnostics, San Antonio, TX, USA) in PBS) was added for 1 hour and left at room temperature. Primary CC-3 antibodies (Rabbit; anti-CC3; 1:500; Cell Signaling Technology, Danvers, MA, USA, Catalogue no. ♯9664) were diluted in blocking solution and added in 30 μL per well and left overnight at 4°C. The following day, primary antibody solutions were

removed and washed twice with phosphate buffered saline (PBS). Wells were then incubated for 30 minutes at room temperature in blocking solution. Following this, secondary antibodies (Alexa 555 goat anti-rabbit, Invitrogen, 1:500) diluted in 30 μL of blocking solution were added and allowed to incubate at room temperature for 2 hours. Wells were then washed twice with PBS and 50 μL 4',6-diamidino-2-phenylindole (DAPI; 1:2000; Sigma) was added per well (for nuclear staining). Wells were then washed three times with PBS and stored in a final third wash at 4°C in preparation for immunofluorescence detection. The CellInsight NXT High Content Screening (HCS) Platform (ThermoScientific, Wilmington, DE, USA) and the Cell Health Profiling Application was used for unbiased quantitative immunofluorescence detection. A representative image of CC-3 staining in shown in Figure 1 below.

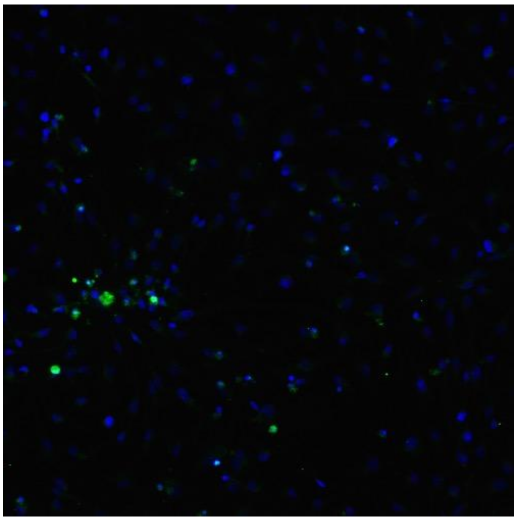

Figure 1: Representative images of CC3 staining shown in green, and DAPI (nuclear) staining shown in blue.

S3: Statistical Analyses for drug toxicity check

For each biological replicate, percentage change in marker immunofluorescence from our drug treated cells relative to controls was calculated for each technical triplicate and the mean was taken. Linear regressions were then used to assess the relationship between percentage change in marker immunofluorescence and: (i) drug/no drug (dose groups collapsed), and (ii) drug dose, whilst co-varying for staining batch. We consider p-values of $P \leq 0.05$ as significant.

S4: Results from drug toxicity checks

Regression analyses revealed that CC3 was not significantly affected by the drug escitalopram, independent of dose ($F = 0.732$, d.f. = 1, $p = 0.425$), or dose-dependently ($F=0.0002$, d.f. = 1, $p = 0.989$). Similarly, CC3 was not affected by nortriptyline, independent of dose ($F = 0.054$, d.f. = 1, $p = 0.823$) or dose-dependently ($F = 1.954$, d.f. = 1, $p = 0.2$).

S5: RNA Quality and Microarray Array Preparation

All RNA samples had 260/280 ratios of above 1.75, as assessed using the Nanodrop ND1000 (Thermoscientific). RNA integrity numbers (RINs) were assessed using the Agilent Bioanalyzer (Agilent Technologies, Berkshire, UK) and all samples had RINs of greater than 9. The current study utilised six biological replicates at four drug doses (n=24 total per drug). RNA samples were processed on Illumina Human HT-12 v4 Expression BeadChip (Illumina Inc., San Diego, CA) according to manufacturer’s protocol. Complementary DNA was synthesized using 300ng of total RNA followed by amplification and

biotinylation of complementary RNA and hybridization according to the protocol supplied with the Illumina Total-Prep RNA Amplification Kit (Life Technologies, UK).

S6: Microarray Preprocessing

Initial quality control assessment was performed in Genome Studio, where outliers were identified using a scatterplot of average signal intensities; no outliers were identified. The Lumi (Bioconductor) package in R (http://www.R-project.org) was used for quality control, quantile-normalization, log-transformation and gene annotation. Genes were then filtered based on detection values generated by Genome Studio. Expression probes had to reach the detection p-value threshold <0.01 in at least one sample, and if not, they were excluded. We further filtered out any probes showing little to no variation across each condition in order to reduce the burden of multiple testing correction, utilizing only the 7,500 most variable probes per group.
